# Genetic association analysis of microRNA137 and its target complex 1 with schizophrenia in Han Chinese

**DOI:** 10.1038/s41598-017-15315-7

**Published:** 2017-11-08

**Authors:** Weihong Lu, Yi Zhang, Xinyu Fang, Weixing Fan, Wei Tang, Jun Cai, Lisheng Song, Chen Zhang

**Affiliations:** 1 Schizophrenia Program, Shanghai Mental Health Center, Shanghai Jiao Tong University School of Medicine, Shanghai, China; 2 Department of Psychiatry, Jinhua Second Hospital, Jinhua, Zhejiang, China; 3 Department of Psychiatry, Wenzhou Kangning Hospital, Wenzhou, Zhejiang, China

## Abstract

Recent genome-wide association studies (GWAS) have identified a strong association signal of microRNA137 host gene (*MIR137*) with schizophrenia. *MIR137* dysfunction results in downregulation of presynaptic target gene complexin 1 (*CPLX1*) and impairs synaptic plasticity in the hippocampus. In this study, we aimed to investigate whether the variants of *MIR137* and *CPLX1* confer susceptibility to schizophrenia in Han Chinese. This study employed 736 patients with schizophrenia patients and 751 well-matched healthy subjects for genetic analysis, and genotyped 12 SNPs within *MIR137* and *CPLX1*. SZDB database was used to performed brain eQTL analysis. There were no significant differences of *CPLX1* expression in hippocampus, prefrontal cortex or stratum between the schizophrenia patients and control subjects. No significant differences were observed in allele and genotype frequencies in studied SNPs between the case and control groups. Gene interaction analysis showed that *MIR137* SNP rs1625579 did not affect schizophrenia susceptibility in interaction with the *CPLX1* polymorphic variants. Our findings do not support *MIR137* and *CPLX1* conferring susceptibility to schizophrenia in Han Chinese.

## Introduction

Schizophrenia is a severe and disabling mental illness with clinical symptoms typically manifesting in a late adolescence or early adulthood onset. Although its etiology and pathophysiology remain unknown, the underlying cause of schizophrenia is suspected to a disruption of early brain development resulted from genetic predisposition and prenatal/perinatal environment factors^1^. A variety of genetic risks identified in schizophrenia are genes expressing proteins involved in the regulation of synaptic plasticity^2^.

Recent genome-wide association studies (GWAS) have identified a strong association signal of microRNA137 host gene (*MIR137*) with schizophrenia^3–5^. MicroRNAs (miRNAs) are small noncoding single-stranded RNAs that function as post-transcriptional regulators of gene expression^6^. In the central nervous system, miRNAs may play an important role in neurodevelopment and maturation including synaptic development, dendritic protein synthesis and neural plasticity^7^. MicroRNA137 is a brain-enriched miRNA in human with high expression in cortical brain regions and hippocampus, and has a critical regulatory role in brain function^8–10^. At the molecular level, a single nucleotide polymorphism (SNP) rs1625579 in *MIR137* has been reported to confer susceptibility to schizophrenia in populations of European ancestry. However, the association of rs1625579 with schizophrenia is inconsistent in Asian populations^11–17^. Pu and Xiao^18^ thereby performed a meta-analysis and provided unsupportive evidence for the association of rs1625579 with schizophrenia in Asians.

A recent study has pointed out that *MIR137* dysfunction results in downregulation of presynaptic target gene complexin-1 (*CPLX1*) and impairs synaptic plasticity in the hippocampus *in vitro* and *in vivo* ^19^. Complexin has a regulatory role in synaptic vesicle exocytosis^20^ and complexin 1 modulates vesicle release^21^. A postmortem study reported that patients with schizophrenia have a significant decrease of CPLX1 protein in prefrontal cortex, when compared with healthy subjects^22^. However, an early genetic study scanned the haplotype-tagging^23^ SNPs in *CPLX1* in a small sample of Japanese patients with schizophrenia, whereas no significant association of *CPLX1* with schizophrenia was observed^24^. As abovementioned, *CPLX1* is downregulated by miRNA137 gain of function, causing impairment in synaptic vesicle trafficking and alterations in synaptic plasticity^19^. Therefore, we hypothesized that the potential interaction effect of *MIR137* and *CPLX1* may influence the genetic risk for schizophrenia.

In this study, we aimed to investigate whether the variants of *MIR137* and *CPLX1* confer susceptibility to schizophrenia in Han Chinese. Here, we first used a public database to detect whether *CPLX1* differentially expressed in brain between patients with schizophrenia and healthy controls. Second, we totally genotyped twelve SNPs of *MIR137* and *CPLX1* in our samples. Meanwhile, we also detected the effect of the two genes interaction in the susceptibility of schizophrenia, because a specific individual genetic variant has a minor marginal effect in such a complex psychiatric disease and gene-gene interaction has importance to describe such effect^25^.

## Results

**Figure 1:**
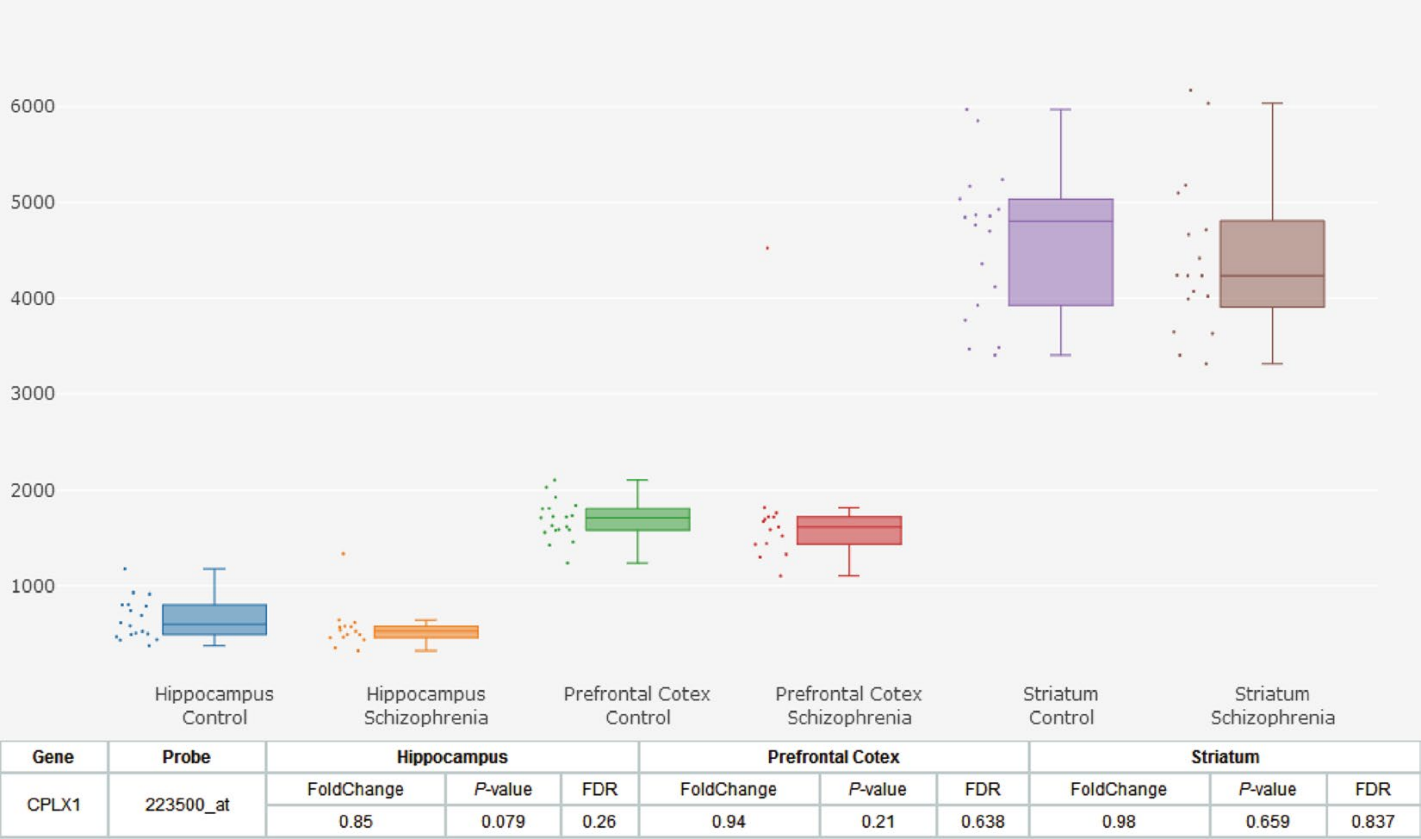
Differential expression of *CPLX1* in brain between patients with schizophrenia and healthy controls. Each bar represents the average level of *CPLX1* expression. Error bars represent the standard deviation of the mean value. Data was extracted from the SZDB database (http://www.szdb.org/).

We extracted brain *CPLX1* expression data between schizophrenia patients and healthy controls from SZDB database^26^. Figure 1 showed that there were no significant differences of *CPLX1* expression in hippocampus, prefrontal cortex or stratum between the schizophrenia patients and control subjects (corrected *P* = 0.26, 0.64, 0.84, respectively).

None of the genotypic distributions showed deviation from the Hardy-Weinberg equilibrium. There were no significant differences in allele and genotype frequencies in any SNP of either *MIR137* or *CPLX1* between the case and control groups (Table 1). After calculating LD for all pairs of SNP markers in *CPLX1*, we found two strong LDs between rs11248043 and rs7376690, as well as rs6832751 and rs10155482 (Supplementary Figure S1). Supplementary Table S3 listed all *P* values corresponding to haplotypes, with rare haplotypes (<3%) being dropped. The haplotypes showed no significant association with schizophrenia. Next, we extracted the schizophrenia genetic association data from the PGC database and observed no significant association of *CPLX1* with schizophrenia either (Supplementary Figure S2). As shown in Table 2, we did not find the *MIR137* SNP rs1625579 affecting schizophrenia susceptibility in interaction with the *CPLX1* polymorphic variants (*P*s > 0.05).

**Table 1:** Comparison of allele and genotype frequencies of the selected SNPs within *MIR137* and *CPLX1* between schizophrenia and healthy control groups.

SNP ID	Genotype	Number of samples		*P*-value^a^	*P*-value^b^	Allele	Number of samples		*P*-value^a^	*P*-value^c^
*MIR137*		Case	Control				Case	Control		
rs1625579	GG/GT/TT	0/94/642	0/85/666	0.39		G/T	94/1378	85/1417	0.40	0.10
*CPLX1*										
rs2242237	CC/CT/TT	38/263/435	46/252/453	0.55		C/T	339/1133	344/1158	0.93	0.17
rs2306251	GG/GA/AA	56/292/388	65/282/404	0.60		G/A	404/1068	412/1090	0.99	0.12
rs11722977	AA/AC/CC	185/355/196	177/396/178	0.21		A/C	725/747	750/752	0.71	0.13
rs7677766	AA/AG/GG	74/290/372	63/339/349	0.07		A/G	438/1034	465/1037	0.48	0.13
rs17165034	AA/AG/GG	38/233/465	31/224/496	0.42		A/G	309/1163	286/1216	0.18	0.37
rs9328758	CC/CT/TT	181/343/212	176/369/206	0.62		C/T	705/767	721/781	0.95	0.67
rs11248042	CC/CT/TT	111/359/266	114/333/304	0.18		C/T	581/891	561/941	0.23	0.15
rs11248043	AA/AG/GG	78/357/301	114/327/310	0.02	0.24	A/G	513/959	555/947	0.23	0.07
rs7376690	AA/AG/GG	301/325/110	313/327/111	0.95		A/G	927/545	953/549	0.79	0.10
rs6832751	AA/AG/GG	28/234/474	23/215/513	0.26		A/G	290/1182	261/1241	0.10	0.93
rs10155482	CC/CA/AA	37/224/475	35/226/490	0.93		C/A	298/1174	296/1206	0.71	0.18

^a^Raw *P*-values. ^b^ *P*-values were calculated after Bonferroni correction. ^c^Hardy-Weinberg *P*-values in the control group.

**Table 2:** Gene-interaction of *MIR137* with *CPLX1* between schizophrenia and healthy control groups.

SNP set		Case interaction	Control interaction	*P*-value^a^	*P*-value^b^
*MIR137*	*CPLX1*				
rs1625579	rs2242237	−0.002	−0.001	0.59	1.00
	rs2306251	−0.002	−0.0003	0.31	1.00
	rs11722977	−0.00006	−0.004	0.11	1.00
	rs7677766	−0.003	−0.001	0.37	1.00
	rs17165034	−0.001	−0.00007	0.49	1.00
	rs9328758	−0.0007	−0.0004	0.97	1.00
	rs11248042	−0.002	−0.008	0.11	1.00
	rs11248043	−0.0004	−0.003	0.05	0.68
	rs7376690	−0.00008	−0.0002	0.82	1.00
	rs6832751	−0.0007	−0.0009	0.65	1.00
	rs10155482	−0.001	−0.001	0.72	1.00

^a^Raw *P*-values. ^b^ *P*-values were calculated after False Discovery Rate (FDR).

On the basis of the genotype data, the statistical power of all SNPs was more than 80% (α = 0.05) for our samples under the assumption of a modest effect size (OR = 1.5) and a log additive model and the disease prevalence of 1%.

## Discussion

In this study, our results did not support the involvement of *MIR137* and *CPLX1* in the pathophysiology of schizophrenia, at least in Han Chinese population. Although a recent meta-analysis showed that *MIR137* SNP rs1625579 significantly increases the risk of schizophrenia^27^, another meta-analysis indicated that the association of rs1625579 with schizophrenia did not exhibit in Asian ancestry resulted from potential genetic heterogeneity between European and Asian populations^18^. Our results provided further evidence to support this conclusion. On the other hand, we failed to find any positive association signals between *CPLX1* and schizophrenia in Han Chinese. This is in line with early literature that Kishi *et al*.^24^ scanned *CPLX1* in Japanese population and observed negative association between *CPLX1* and schizophrenia. Given the modulatory effect of microRNA137 on complexin 1^19^, we hypothesized a gene interaction between the *MIR137* and *CPLX1* may confer susceptibility to schizophrenia. However, our explorative analysis did not support this hypothesis.

A recent postmortem study found that transcript level for *CPLX1* is significantly decreased in the anterior cingulate cortex (ACC) of schizophrenia patients^28^, whereas our results showed *CPLX1* expression does not alter in hippocampus, prefrontal cortex or stratum of schizophrenia patients. It is generally accepted that ACC contributes to cognitive control, decision-making, empathy and emotion^29,30^. Animal experiment showed that *CPLX1* knockout mice have pronounced deficits in social behaviors^31^. It is known that schizophrenia is characterized by persistent cognitive deficits, positive and negative symptoms and its etiological heterogeneity is manifested^32–35^. Therefore, although no association of *CPLX1* with schizophrenia susceptibility was observed in our samples, we could not fully exclude the possible involvement of *CPLX1* in the development of cognitive dysfunction in schizophrenia.

MicroRNA137 is enriched in hippocampal and cortical neurons that play important roles in neuronal maturation and dendritic spine morphogenesis^36^. It is known or predicted to regulate hundreds of genes, whose targets include many schizophrenia susceptibility genes, such as *BDNF*, *ZNF804A*, *TCF4* and *CACNA1C* ^37,38^. Therefore, *MIR137* associated risk for schizophrenia may be implicated with its downstream genetic effects^38^. Although we did not find the evidence for the involvement of *MIR137* and *CPLX1* in schizophrenia, further investigations are required to detect the interplay of *MIR137* with its target genes in the susceptibility to schizophrenia.

This study has several limitations that should be taken into account. First, this is an exploratory study performed on a subset of the general Chinese Han population. The sample size is modest and precludes us from making any definitive statements on the associations of *MIR137* and *CPLX1* with schizophrenia in Han Chinese. Second, cross-sectional association studies always have the potential for population stratification. In this study, our samples were collected from Eastern China and may not be representative of the Han Chinese population in general, nor other closely related populations in the area. Third, this study was designed based on the “Common Disease-Common Variant” hypothesis, and we did not sequence the genes to assess the influence of more rare variant(s) on schizophrenia. Future targeted deep sequencing may help to undercover fundamental characteristics of pathogenic *MIR137* and *CPLX1* mutations and any potential association with schizophrenia.

In conclusion, our findings do not support *MIR137* and *CPLX1* conferring susceptibility to schizophrenia in Han Chinese. Further investigations are warranted to validate our results and identifying the polygenic effects of *MIR137* with its downstream target genes in the pathophysiology of schizophrenia.

## Methods

### Subjects

All procedures were reviewed and approved by Institutional Review Boards of Shanghai Mental Health Center and other participating institutions. This study was performed in accordance with the guidelines laid out in the Declaration of Helsinki as revised in 1989. All subjects provided written informed consent before any study-related procedures were performed.

A total of 736 schizophrenia patients were recruited three mental hospitals in Eastern China, including Shanghai Mental Health Center, Shanghai Jiao Tong University School of Medicine, Jinhua Second Hospital and Wenzhou Kangning Hospital. The inclusion criteria for this study were according to our previous publications^16,39,40^. All patients (1) met the Diagnostic and Statistical Manual of Mental Disorders, Fourth Edition (DSM-IV) criteria for schizophrenia; (2) were not first-episode; (3) had no chronic physical disease or other psychiatric disorder aside from schizophrenia. Prior to analysis, all diagnosis and review of psychiatric case records were independently checked and verified by two senior psychiatrists. The schizophrenia patients were matched with 751 control subjects enrolled from the hospital staff and students of the School of Medicine in Shanghai, all of which were interviewed by a specialized psychiatrist using the Structured Clinical Interview for DSM-IV-TR Axis I Disorders-Patient Edition (SCID-P) to determine that they had no psychiatric disorders^34,41^. Any healthy controls found to have any psychiatric disorder or chronic physical disease were excluded from this analysis. The patient and control groups were matched demographically, except education. Detailed participant information was summarized in Supplementary Table S1. All subjects in both the patient and control group were of Han Chinese origin.

### SNP selection

We retrieved CHB data from the HapMap database (http://www.hapmap.org) and defined linkage disequilibrium (LD) blocks using Haploview 4.2 (Broad Institute, Cambridge, MA, USA) to set inclusion criteria for tagging SNPs. Haplotype-tagging single nucleotide polymorphisms (htSNPs) with *r* ^2^ cutoff >0.8 and minor allele frequency (MAF) >0.1 were selected. In total, eleven tag SNPs of *CPLX1* were captured for genotyping, including rs2242237, rs2306251, rs11722977, rs7677766, rs17165034, rs9328758, rs11248042, rs11248043, rs7376690, rs6832751 and rs10155482 (Supplemental Table S2).

### Genotyping

Genomic DNA of all participants was extracted from peripheral blood using a Tiangen DNA Isolation Kit (Tiangen Biotech, Beijing, China). SNP rs1625579 in *MIR137* and 11 htSNPs in *CPLX1* were genotyped with the Improved Multiplex Ligase Detection Reaction (iMLDR) method described in our previous study^42^, with technical support from the Center for Human Genetics Research, Shanghai Genesky Biotech Co., Ltd. The technicians performing genotyping were blind to the study participants. Ten percent of the samples were later randomly selected for duplicate genotyping, which produced 100% concordance.

### Brain eQTL analysis for CPLX1 expression

It is known that schizophrenia originates from brain structural and functional abnormalities^43,44^, and dysregulation of gene expression has a key role in the pathogenesis of this disease. In this study, we performed an eQTL analysis to detect whether *CPLX1* is differentially expressed in brain between patients with schizophrenia and healthy controls, using SZDB database (http://www.szdb.org/), a newly developed comprehensive resource for schizophrenia research^26^.

### PGC data analysis

To further validate the association between the *CPLX1* and schizophrenia, we extracted the schizophrenia genetic association data from the Psychiatric Genomics Consortium (PGC, http://www.broadinstitute.org/mpg/ricopili/) database^4^ and reanalyzed the data set as an independent sample.

### Statistical analysis

Demographic data were analyzed using chi-squared or *t*-test as appropriate. Single marker and gene interaction analyses were conducted using SHEsisPlus (http://shesisplus.bio-x.cn/)^45,46^. The level of significance was corrected for multiple testing. Pair-wise linkage disequilibrium (LD) and haplotype analyses were calculated using Haploview 4.2, and the extent of LD was measured by the standardized *D*’. The haplotypes with a frequency under 3% were ignored. Power analysis was performed using Quanto 1.2.3 (http://hydra.usc.edu/GxE). All statistical analyses were carried out by using the SPSS 17.0 (SPSS, Inc., Chicago, IL, USA). Criterion for statistical significance was set at *α* = 0.05 and results were two-tailed.

## Electronic supplementary material

Supplementary Information
