# Supplementary material for: Genetic association analysis of microRNA137 and its target complex 1 with schizophrenia in Han Chinese
Source: Sci Rep. 2017 Nov 8;7:15084. doi: 10.1038/s41598-017-15315-7 (PMC5678134; doi:10.1038/s41598-017-15315-7)
Supplement: Supplementary file 1 — Supplementary Information [file 41598_2017_15315_MOESM1_ESM.doc]

**Genetic association analysis of microRNA137 and its target complex 1 with schizophrenia in Han Chinese**

Weihong Lu^a^, Yi Zhang^a^, Xinyu Fang^a^, Weixing Fan^b^, Wei Tang^c^, Jun Cai^a^, Lisheng Song^a^, Chen Zhang^a^,*

^a^ Schizophrenia Program, Shanghai Mental Health Center, Shanghai Jiao Tong University School of Medicine, Shanghai, China

^b^ Department of Psychiatry, Jinhua Second Hospital, Jinhua, Zhejiang, China

^c^ Department of Psychiatry, Wenzhou Kangning Hospital, Wenzhou, Zhejiang, China

Running title: microRNA137, complex 1 and schizophrenia

* Corresponding authors

Chen Zhang, Email: [zhangchen645@gmail.com](mailto:zhangchen645@gmail.com) (C. Zhang)

Supplementary Table S1 Demographic characteristics of case and control groups

|  | Case (n=736) | Control (n=751) | χ^2^/t | *P* |
| --- | --- | --- | --- | --- |
| Sex (M/F) | 436/300 | 473/278 | 2.2 | 0.15 |
| Age (years) | 33.8±8.9 | 33.0±8.3 | 1.8 | 0.08 |
| Education (years) | 10.8±1.4 | 13.1±2.4 | -23.4 | ＜0.01 |
| Age at onset (years) | 25.3±3.3 |  |  |  |
| Duration of illness (years) | 8.5±7.7 |  |  |  |

Note: M/F, male/female

Supplementary Table S2 Information of selected SNPs genotyped in this study

| SNP | Chromosome | Allele | Position | Function |
| --- | --- | --- | --- | --- |
| *MIR137* |  |  |  |  |
| rs1625579 | 1 | T/G | 98037378 | intron |
| *CPLX1* |  |  |  |  |
| rs2242237 | 4 | C/T | 785606 | upstream |
| rs2306251 | 4 | A/G | 792613 | intron |
| rs11722977 | 4 | A/C | 796969 | intron |
| rs7677766 | 4 | A/G | 802102 | intron |
| rs17165034 | 4 | A/G | 805174 | intron |
| rs9328758 | 4 | C/T | 815999 | intron |
| rs11248042 | 4 | C/T | 818810 | intron |
| rs11248043 | 4 | A/G | 818965 | intron |
| rs7376690 | 4 | A/G | 819938 | intron |
| rs6832751 | 4 | A/G | 820292 | intron |
| rs10155482 | 4 | A/C | 823133 | intron |

Supplementary Table S3 Results of the *CPLX1* pairwise haplotype test between case and control groups

| Haplotype^a^ | Frequency (%) | |  |
| --- | --- | --- | --- |
|  | cases | controls | *P* ^b^ |
| rs11248043-rs7376690 |  |  |  |
| G-G | 36.7 | 36.2 | 0.80 |
| A-A | 34.5 | 36.6 | 0.23 |
| G-A | 28.5 | 26.8 | 0.31 |
| rs6832751-rs10155482 |  |  |  |
| G-A | 60.1 | 63.1 | 0.09 |
| G-C | 20.2 | 19.6 | 0.66 |
| A-A | 19.7 | 17.3 | 0.10 |

^a^ Haplotypes with frequency ＜3% are ignored in analysis.

^b^ *P* values for single haplotype test, d.f.=1, not corrected for multiple test.

Supplementary Figure S1 Linkage disequilibrium plots consisting of 11 SNPs within *CPLX1* in schizophrenia. Pairwise linkage disequilibrium (LD) was computed for all possible combinations using the values of *D*’ and *r*^2^.

*D*’ *r*^2^

Supplementary Figure S2 Association of *CPLX1* with schizophrenia in PGC database
